# Potential role of the lncRNA "HOTAIR"/miRNA "206"/BDNF network in the alteration in expression of synaptic plasticity gene arc and BDNF level in sera of patients with heroin use disorder through the PI3K/AKT/mTOR pathway compared to the controls

**DOI:** 10.1007/s11033-024-09265-3

**Published:** 2024-02-09

**Authors:** Fatma Nada Khalifa, Riham F. Hussein, Dina M. Mekawy, Heba M. Elwi, Shimaa Ahmed Alsaeed, Yassmin Elnawawy, Somaya H. Shaheen

**Affiliations:** 1 Department of Forensic Medicine and Clinical Toxicology, Faculty of Medicine, Cairo University, Kasr Alainy Street, Cairo, 11562 Egypt; 2 Department of Biochemistry, Faculty of Medicine, Cairo University, Kasr Alainy Street, Cairo, 11562 Egypt; 3 Department of Psychiatry, Faculty of Medicine, Cairo University, Kasr Alainy Street, Cairo, 11562 Egypt

**Keywords:** Heroin abuse, mTOR, BDNF, miRNA-206, HOTAIR, Expression

## Abstract

**Introduction:**

Heroin use disorder (HUD) is a seriously increasing health issue, accounting for most deaths among drug abusers. Studying non-coding ribonucleic acid gene expression among drug abusers is a promising approach, as it may be used in diagnosis and therapeutics.

**Participants and methods:**

A total of 49 male heroin-dependent patients and 49 male control participants were recruited from Kasr Al Ainy Psychiatry and Addiction outpatient clinics, Faculty of Medicine, Cairo University. Sera were gathered. qRT-PCR was utilized for the detection of gene expression of non-coding RNAs such as "HOX transcript antisense RNA" (HOTAIR), micro-RNA (miRNA-206), phosphatidylinositol 3-kinase (PI3K), protein kinase B (AKT), mechanistic target of rapamycin (mTOR), and Activity Regulated Cytoskeleton Associated Protein (Arc). Sera Brain-Derived Neurotrophic Factor (BDNF) levels were assessed using ELISA. Using a western blot made it possible to determine the protein expression of PI3K, AKT, and mTOR.

**Results:**

The study demonstrated that gene expressions of HOTAIR, AKT, PI3K, and Arc were considerably lowered between cases and controls, while gene expressions of miR-206 and mTOR1 were significantly raised. PI3K and AKT protein expressions were downregulated, while mTOR expressions were upregulated. BDNF levels were significantly decreased in some cases.

**Conclusion:**

The results of this study suggest that decreased HOTAIR in HUD relieves miR-206 inhibition, which thus increases and affects downstream PI3K/AKT/mTOR, ARC, and BDNF expression. This may be shared in addictive and relapsing behaviors.

**Graphical Abstract:**

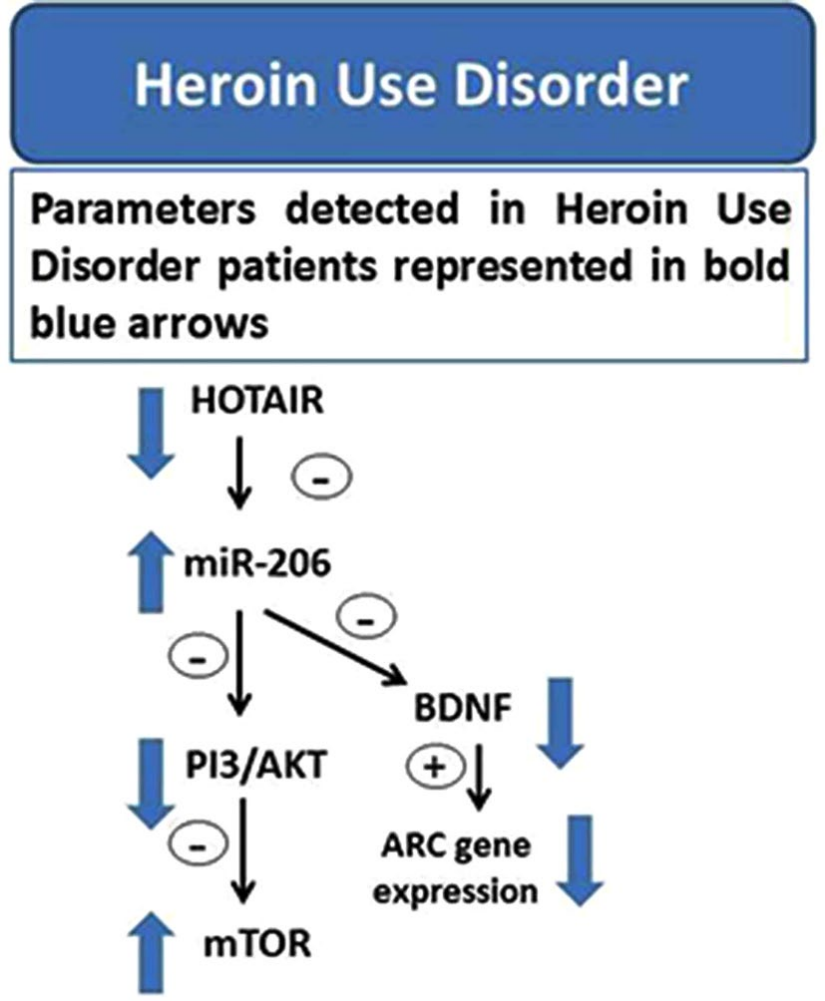

## Introduction

Substance use disorder (SUD) is a social issue that affects communities worldwide. Since the 1970s, there has been a significant increase in the prevalence of SUD in Egypt [1]. The World Health Organization **(WHO)** asserts that [2]. The prevalence of drug addiction among Egyptians aged 15–64 years old is 0.8%, of which 44% pertains to opioids [1]. Heroin is a highly addictive opioid [3]. Heroin use disorder (HUD) heavily impacts the socio-economic status of the country [4].

Available pharmacotherapies focus on the modulation of neurotransmitter receptors [5]. This approach demonstrates clinical success, yet some therapy-related issues must be addressed to formulate a plan that prevents relapses. Therapeutic opportunities targeting non-coding ribonucleic acids (ncRNAs) could be explored, opting for their tissue-specific regulatory impact on many biological processes, including synaptic plasticity [6–8].

Several studies questioned the relationship between HUD and ncRNAs, mostly long ncRNA (lncRNA) and microRNAs (miRNAs)**.** Therapeutic injection of anti-miRNAs and miRNA mimics targeting miRNAs implicated in the pathogenesis of SUD has been tried in animal models of opioid use disorder (OUD) [9]. Particular attention should be given to the identification of ncRNAs involved in relapses and plasticity to promote abstinence and long-term recovery in humans.

Short non-coding RNAs (ncRNAs), called miRNAs, regulate gene expression via cleavage of messenger RNA (mRNA) or inhibition of translation. [10]. They have significant roles in the long-term neuroadaptations related to drug abuse [11]. Gu et al. discovered a substantial increase in serum miR-206 levels among heroin drug abusers [12]. Moreover, miR-206 was previously found to contribute to alcohol dependence by reducing BDNF mRNA production, a crucial molecule implicated in plastic changes associated with memory and learning [13].

Long non-coding RNAs (lncRNAs) are 200 + nucleotide transcripts that regulate gene expression at the epigenetic, transcriptional, and posttranscriptional levels. Various studies have demonstrated that lncRNAs, like HOX transcript antisense RNA (HOTAIR), have a significant impact on normal neural development and addiction [14]. Interestingly, HOTAIR was previously found to regulate miR-206 by sponging it [15–18].

Human brain BDNF is the neurotrophin with the most outstanding distribution. In addition, it governs synaptic neurotransmission, neural plasticity, neuronal survival, function, and development [13]. The pathophysiology of heroin addiction and withdrawal syndrome has been linked to BDNF, as previous studies found that heroin users have lower serum BDNF levels [19] despite having higher serum BDNF levels during early abstinence [20]. BDNF activates several pathways, including phosphatidylinositol 3 (PI3K/AKT) via tyrosine kinase B activation (TrKB) [21].

The mechanistic goal of rapamycin complex 1 (mTORC1) dysfunction has been reported in addiction [22]. Upstream PI3/AKT is known to inhibit mTORC1. A report by Zhu et al. 2021 revealed the possible function of the PI3K/AKT/mTOR signaling pathway in the process of heroin addiction and relapse since they revealed an essential function of nucleus accumbens (NAc) AKT phosphorylation in the incubation of heroin-seeking behavior [23].

The activity-regulated cytoskeleton-associated protein **(**Arc) encodes the Arc/Arg3.1 protein, a primary regulator of synaptic plasticity that autonomously assembles into virion-like capsids, facilitating intercellular RNA transfer and encapsulating RNAs within the nervous system. [9, 24]. Arc was found to be an essential effector of BDNF [10].

This research aims to evaluate and correlate the expression levels of HOTAIR and miR-206 in HUD. It is known that HOTAIR expression has never been measured in HUD. In addition, it aims to detect how HOTAIR and miR-206 may affect the expression of synaptic plasticity genes Arc and BDNF by exploring the expression of the PI3-AKT-mTOR pathway.

## Participants and procedures

### Participants

Forty-nine masculine heroin-dependent patients were recruited from Kasr Al-Ainy Psychiatry and Addiction outpatient clinics, Faculty of Medicine, Cairo University. The patients were diagnosed with heroin dependency following the *Diagnostic and Statistical Manual of Mental Disorders* (DSM-IV) (age 18–50 years). All heroin-dependent participants were current and untreated heroin users. Patients with any substance use disorder other than heroin (except smoking) or significant psychiatric disease and those with chronic medical illnesses (neurological, renal, hepatic, metabolic, endocrine, cardiac, autoimmune, or respiratory disorders) were excluded from the study.

The enrolled control participants (n = 49) matched the patient group and underwent the same scales and tools. The control group participants had no history of drug abuse. Participants with psychiatric, neurological, hepatic, or renal diseases were excluded.

### Methods

Written approval from all participants was collected. The Ethical and Research Committee of Kasr Al-Ainy, Cairo University, accepted this research. The demographic and clinical data of the participants (name, education, age, occupation, heroin use relapses, the age at which heroin use begins, and duration and dosage of heroin) were recorded and evaluated. The heroin dependence diagnosis was performed utilizing the Structured Clinical Interview for DSM-IV, which is a clinician-administered, semi-structured interview that is used in patients with psychiatric diseases and in healthy participants who are undergoing psychiatric assessment.

All eligible participants were subjected to a complete history taking, clinical assessment, and urine tests for psychoactive substances to exclude or confirm their use of opiates and other substances other than opiates. Blood samples were collected in heparinized tubes from every participant who fulfilled the inclusion criteria and sent to the Biochemistry Department, Faculty of Medicine, Cairo University, for analysis and quantitative determination of HOTAIR, miR-206, PI3K, AKT, mTOR, and Arc using the quantitative real-time polymerase chain reaction (qRT-PCR) technique. ELISA was utilized to measure BDNF protein amounts in sera.

### Blood specimens

A volume of around 5 mL of venous blood was collected from each participant utilizing the BD Vacutainer system. After collecting the samples in serum separator containers, they were centrifuged at 4000 × g for 10 min after being allowed to thicken for 15 min. After being aliquoted, sera were maintained at −80 °C until analysis commenced.

### Extraction of RNA

Aliquots of one hundred μL of serum were used for total RNA isolation utilizing SinaPure™ RNA (SinaClon BioScience, Tehran, Iran; Cat. No. EX6031) in accordance with the manufacturer's instructions for purifying serum total RNA, including miRNA. Firstly, four hundred μL of warmed lysis buffer were added to the one hundred μL sera in 1.5 ml tubes. The samples were vortexed for twenty seconds, then three hundred µL Precipitation solution were added. After which, the tubes were inverted ten times. Solutions were then transferred to spin columns within collection tubes and centrifuged at 12,000 g for a minute. Flow-through was discarded. Washing was done twice by the addition of four hundred μL of wash buffer followed by a one-minute centrifugation at 12,000 g, and the disposal of the flow-through. A final two-minute centrifugation was done to ensure the elimination of all fluid. The columns were then transferred into sterile two mL tubes. Fifty µL of 55 °C pre-heated RNase free water was added to the center of the columns. The tubes were then incubated for five minutes at 55 °C. Eventually, centrifugation at 12,000 g for a minute was performed to elute the RNA.

RNA quantification and purity evaluation were performed on the extracted RNA utilizing the NanoDrop® (ND)-1000 spectrophotometer (NanoDrop Technologies, Inc. Wilmington, USA). Blanking the Nano-Drop spectrophotometer was initially done. A spectrum of a reference material (blank) was taken and recorded in memory as an array of light intensities by wavelength. The measurement of samples was recorded. The samples and the blank intensities were used to calculate the sample absorbance considering the following equation: Absorbance =  − log (Intensity sample /Intensity blank). The concentration calculation was automated.

### Reverse transcription of the complementary DNA molecule

Following the manufacturer's instructions, the extracted total RNA in a 20-μL volume of RT reactions was incubated for 60 min at 37 °C, followed by 5 min at 95 °C, using the miScript® II RT Kit (Thermo Fisher Scientific, Vilnius County, Lithuania; Cat. No. 4374966). Until the subsequent procedure, the complementary DNA (cDNA) of each sample was stored at −80 °C.

### Detection of relative gene expressions of RNAs utilizing qRT-PCR

The miScript SYBR® Green chi-miR-206 Assay ID472791_mat PCR Kit (Thermo Fisher Scientific, Germany; Cat. No. 4440886) was used as a portion of the miScript PCR system and target-specific miScript primer analysis for miR-206 and HOTAIR. The profile of thermal cycling was as follows: cDNA synthesis at 45 °C for 15 min is followed by reverse transcriptase inactivation and polymerase activation at 95 °C for 5 min. Following this, 40 PCR amplification cycles were executed, comprising 15 s of DNA denaturation at 95 °C, 20 s of primer annealing at 55 °C, and 30 s of amplification at 72 °C. Table 1 summarizes the primer sequences for all genes. To verify the specific expression of the targeted RNAs, melting curves were examined after PCR cycles. Due to the absence of an endogenous reference housekeeping gene or microRNA in the serum, SNORD-68, a small nucleolar RNA C/D box 68, was utilized as an endogenous housekeeping gene. This choice was taken because SNORD-68's expression remains constant across experimental conditions and states of the same sample, thereby ensuring its reliability. Additionally, the relative quantification of the target miRNAs and validation of the expression pattern were both accomplished with SNORD-68.

**Table 1:** Primer's sequence of the studied genes

Gene symbol	Primer sequence from 5′- 3′ F: Forward primer, R: Reverse primer	Gene bank accession number
lncRNA HOTAIR	F: GGCAGCACAGAGCAACTCTA R: GCAGGGTCCCACTGCATAAT	NR_047518.1
miRNA 206	F: CCGAGGCCACATGCTTCTTTA R: TTGCCGAAACCACACACTTC	NR_029713.1
Arc	F: GGACCCTCGAGAGTTCCTGA R: AACTCCCACCACTTCTTGGC	NM_015193.5
mTOR	F: ATCCAGACCCTGACCCAAAC R: TCCACCCACTTCCTCATCTC	NM_001386500.1
PI3K	F: TCATGCATTGTTTTGCACCCC R: AATGGGATAGTGCCTGAGCC	NM_006218.4
AKT	F: ATTTCCCTCTTTGGGGGCTTAG R: TACTCCCCTCGTTTGTGCAG	NM_001382432.1
G3PD	F: TGGCATATCTCTTATTAAGGGGG R: ACTTCTCATCAGCC ACCTCG	NC_000012.12
SNORD68	F: TGTAAAACGACGGCCAGT R; CAGGAAACAGCTATGACC	NR_028128.1

*lncRNA HOTAIR* long noncoding RNA “HOX transcript antisense RNA”; *miRNA-206* micro-ribonucleic acid; *mTOR* mechanistic target of rapamycin; *PI3K* phosphatidylinositol 3-kinase; *AKT* protein kinase B; *G3PD* glyceraldehyde 3-phosphate dehydrogenase; *SNORD-68* small nucleolar RNA C/D box 68

In addition, glyceraldehyde 3-phosphate dehydrogenase (GAPDH) was utilized as an internal control in the calculations for the long non-coding HOTAIR and the remaining genes. The fold changes in expression of the Arc gene, PI3K, AKT, mTOR, and the long non-coding RNA HOTAIR were subsequently determined using the 2^−ΔΔCt^ method.

### Serum human BDNF estimation by enzyme-linked immunosorbent assay

Serum human BDNF estimation by ELISA (ID SL0371Hu, Sunlong Biotech Co., Hangzhou, China) was performed according to the manufacturer's guidelines. Firstly, Dilution of standards was done in 1.5 mL tubes to a final concentration of: 900 pg/mL, 600 pg/mL, 300 pg/mL, 150 pg/ml, and 75 pg/mL. Then, fifty uL from each standard were added to the microplate wells in duplicates. A well was left empty as a blank control. As for the sample wells, forty μL of the sample dilution buffer were added followed by the addition of ten μL of sera samples. The plate was sealed, gently shaked for proper mixing, and incubated for thirty minutes at 37 °C. Peeling off the plate seal was done carefully. The wells were aspirated and refilled with the wash solution for thirty seconds, then it was discarded. The washing steps were repeated five times. Fifty μL of the HRP-Conjugate were added to each well except the blank control well. The plate was sealed and incubated for thirty minutes at 37 °C. Peeling off the plate seal was done carefully. The wells were aspirated and refilled with the wash solution for thirty seconds, then it was discarded. Coloring was performed by the addition of fifty μL Chromogen Solution A and fifty μL Chromogen Solution B to each well. Gentle shaking and incubation at 37 °C for 15 min were done in a dark place. Fifty μL of the stop solution were added to each well to terminate the reaction. Absorbance O.D. was Read at 450 nm using a Microtiter Plate Reader. The OD value of the blank control well was set as zero.

### Western blot

First, the ReadyPrep™ total protein extraction kit (Bio Rad Inc., California, USA, #163–2086) was performed following the manufacturer's guidelines. The Bradford Protein Assay Kit (Bio Basic Inc., New York, USA, #SK3031) was applied to every sample to quantify protein concentration. Subsequently, 20 μg protein concentration was added to equal volumes of 2 × Laemmli sample buffer (4% SDS, 10% 2-mercaptoethanol, 20% glycerol, 0.004% bromophenol blue, and 0.125 M Tris HCl [pH 6.8]). Protein denaturation was achieved by subjecting the mixture to boiling at 95 °C for 5 min prior to depositing it onto the polyacrylamide gel electrophoresis. In accordance with the manufacturer's guidelines, the SDS-PAGE TGX Stain-Free™ FastCast™ Acrylamide Kit (Bio-Rad Laboratories Inc., Cat # 161–0181) was formulated. After assembling a transfer sandwich consisting of filter paper, PVDF membrane, gel, and filter paper, it was transferred to a transfer vessel containing 1 × transfer buffer (25 mM Tris, 190 mM glycine, and 20% methanol). Protein bands were transferred from the gel to the membrane using BioRad Trans-Blot Turbo during a 7-min blot operation at 25 V. The membrane block was performed for one hour at room temperature in Tris-buffered saline, Tween 20 (TBST) buffer, and 3% bovine serum albumin (BSA) (20 mM Tris pH 7.5, 150 mM NaCl, 0.1% Tween 20, and 3% BSA). Each primary antibody for tPI3K, pPI3K, tAKT, pAKT, tmTOR, and pmTOR was diluted in TBST and incubated overnight against the blotted target protein at 4 °C. Then, 5-min rinses of the blot using TBST were conducted five times.

Following a one-hour incubation at room temperature in the HRP-conjugated secondary antibody solution (Goat anti-rabbit IgG-HRP-1 mg Goat mab-Novus Biologicals), the sample was rinsed five times with TBST for five minutes.

The intensity of the bands was determined using the ChemiDocTM imaging system and Image LabTM software version 5.1 (Bio-Rad Laboratories Inc., Hercules, CA, USA). The outcomes were presented in arbitrary units subsequent to the normalization process for the expression of the β-actin protein.

### Statistical analysis

The statistical analysis was conducted utilizing Version 25 of IBM^1^® SPSS^2^® Statistics. Using the Chi-square test, categorical data were analyzed and presented as frequencies and percentages. The mean and standard deviation (SD) were provided for numerical information. The normality of the data was investigated by examining the data distribution with the Kolmogorov–Smirnov and Shapiro–Wilk tests. For comparisons between two groups, parametric data were analyzed using an independent t-test; the Mann–Whitney U test was utilized for nonnormally distributed data. Pearson's correlation was applied to examine the correlation between variables. In all experiments, a significance level of P ≤ 0.05 was established.

## Results

In this study, the majority (63.3%) of the studied participants are aged 30–50 years old compared with the minority (36.7%) aged 18–30 years old, with a significant statistical difference (*p* < 0.001). Heroin-dependent patients who had primary education have a statistically more considerable proportion than those who had university and postgraduate education (91.8% vs. 8.2% and 0%, *p* < 0.001). Similarly, patients who reported their occupation as manual labor (85.7%) are significantly more than those engaged in mental work (14.3%) (*p* < 0.001) (Table 2).

**Table 2:** Median, mean and the standard deviation for the number of relapses, age of onset, duration of abuse and the amount of heroine usage

Heroin	Minimum	Maximum	Median	Mean	SD
Heroin use relapses	0	13	3	2.65	2.52
Age of onset of heroin use (years)	17	46	25	26	7.25
Duration of heroin use (years)	0.5	30	5	5	5.74
Dose of heroin use (grams)	0.25	5	1	1	1.16

*SD* Standard deviation

The median number of heroin relapses is 3 (0 to 13 relapses reported among study participants), with a mean ± SD of 2.65 ± 2.52. The age at which heroin use begins in study participants is relatively young (mean ± SD = 26 ± 7.25) with a median of 25 (ages ranged from 17 to 46 years old). The mean ± SD of the duration of heroin use is 5 ± 5.74. The minimum duration is six months, and the maximum duration is thirty years. The maximum dose of heroin use in study participants is 5 g/day, while the minimum is 0.25 g, with a median and a mean ± SD of 1 and 1 ± 1.16, respectively (Table 2).

Forty-nine of the study participants used the intravenous route, while 42.8% sniffed heroin, and only 8.2% tried both methods (P value = 0.002). 71.4% of study participants declined the presence of family history as opposed to positive family history in 28.6% of participants (P value: 0.003) (Table 3).

**Table 3:** Methods of heroin use in family history in case group

		Frequency	Percent	p value
method	both	4	8.2	0.002
	i.v	24	49	
	sniff	21	42.8	
Family history	yes	14	28.6	0.003
	no	35	71.4	

P ≤ 0.05 is significant

**Fig. 1:**
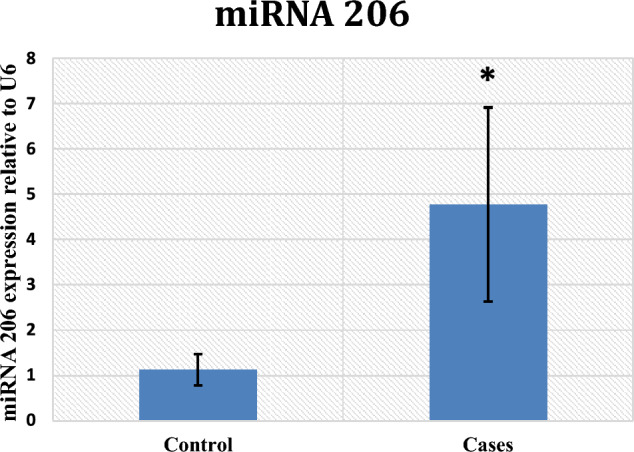
miRNA 206 gene expression among control and cases

**Fig. 2:**
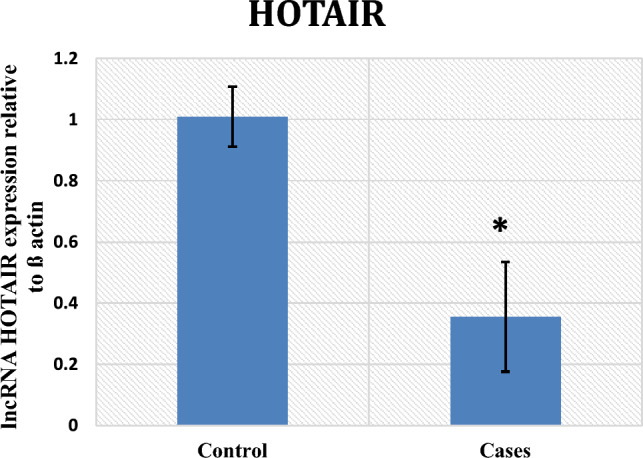
lncRNA HOTAIR expression among control and cases

**Fig. 3:**
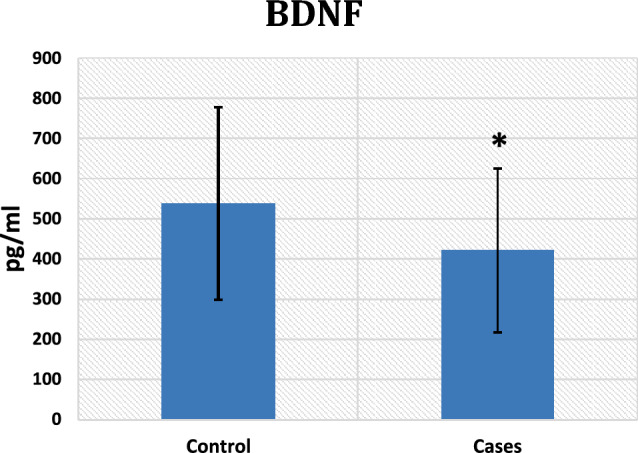
statistically significant downregulation of BDNF among cases. ^*^Denotes significant difference versus controls

In contrast to the control group, the results illustrated a statistically noteworthy increase in miR-206 expression in the patient group with a p-value < 0.001 (Fig. 1), along with a significant downregulation of the expression of HOTAIR (p-value < 0.001) and a substantial decrease in serum levels of BDNF among cases (p-value = 0.01) (Figs. 2 and 3, respectively).

**Fig. 4:**
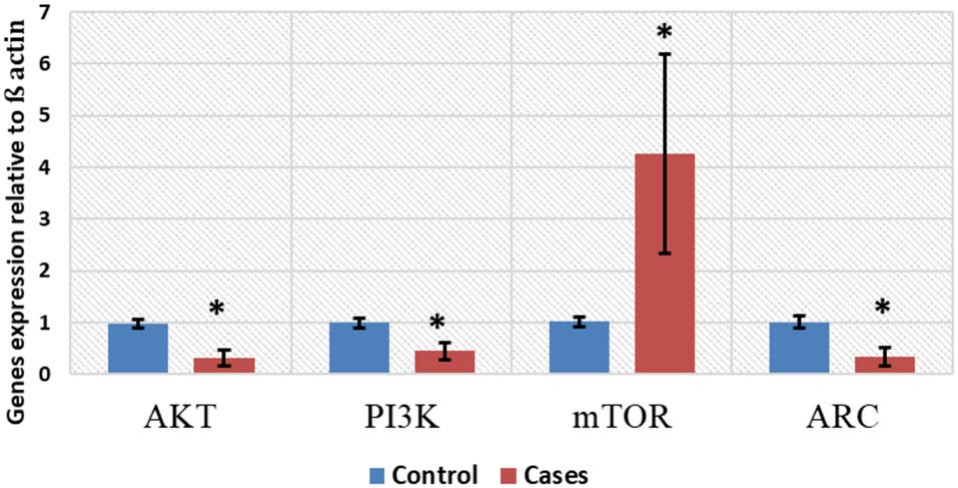
Heroin-dependent patients were compared to participants in the control group who had no prior history of drug abuse. The quantitative real-time polymerase chain reaction (qRT-PCR) method is used for the analysis and quantitative determination of PI3K, AKT, mTOR, and Arc. When comparing cases to controls, there was a statistically significant increase in mTOR expression and a decrease in the expression of AKT, PI3K, and Arc

**Fig. 5:**
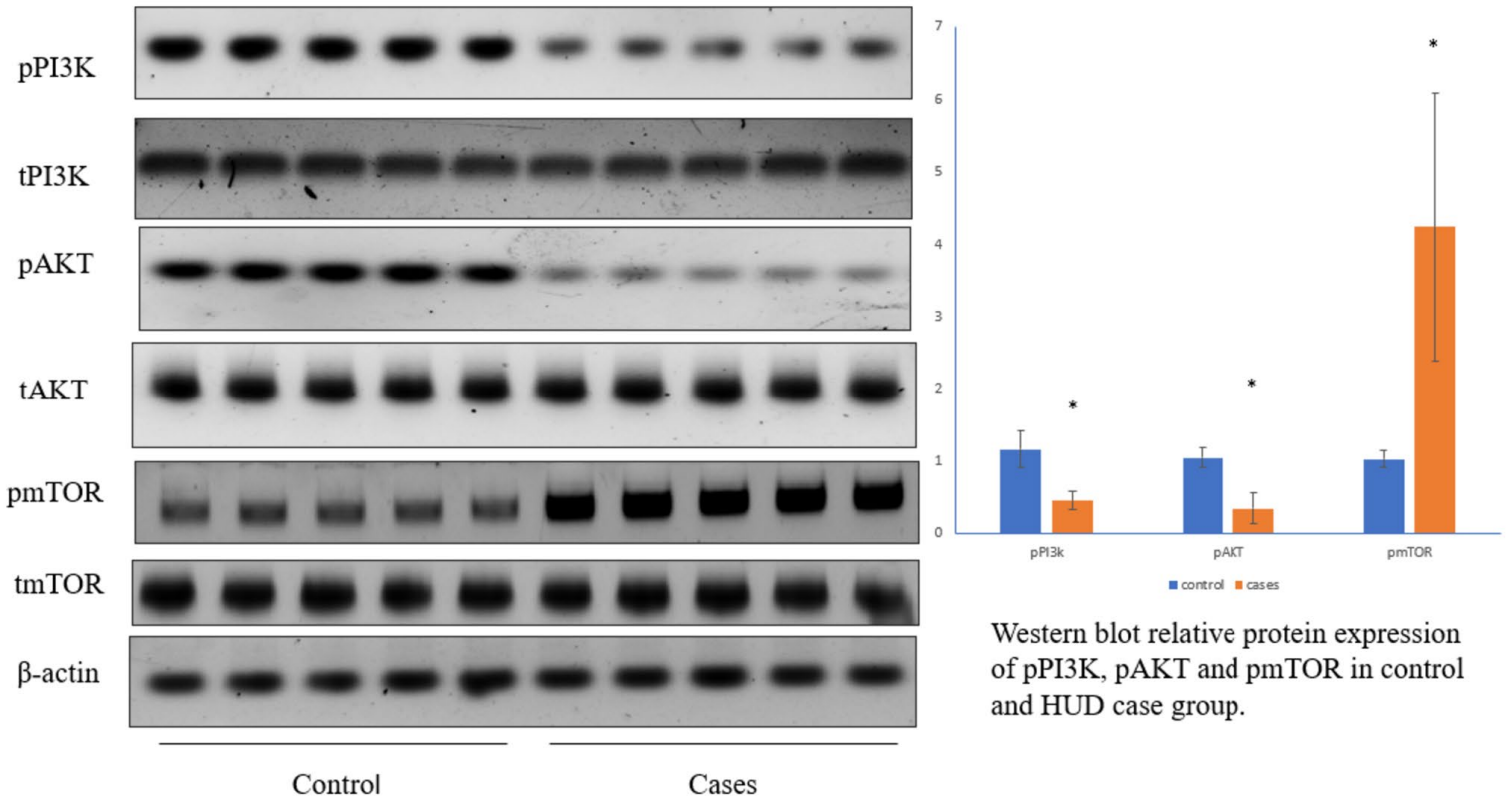
The relative protein expression of phosphorylated and total PI3K, phosphorylated and total AKT, and phosphorylated and total mTOR

Concerning the study of the PI3K/AKT/mTOR pathway, the relative gene expression demonstrated a significant statistical increase in the mTOR expression with a reduction in the AKT as well as PI3K expression levels among cases in comparison to controls (p < 0.001) (Fig. 4). The protein expression coincides with mRNA expression, with underexpression of phosphorylated PI3K and phosphorylated AKT, along with overexpression of mTOR in some cases (p < 0.001) (Fig. 5). In addition, the relative gene expression of the synaptic plasticity gene 'Arc' was meaningfully reduced in cases in comparison with the control group (p-value = 0.01) (Fig. 4).

## Discussion

Long-term heroin use causes variations in gene and protein expression in the brain. This is linked to activity-dependent synaptic plasticity, which results in a higher risk of drug-seeking behavior and heroin relapses. The molecular mechanisms that underlie substance use disorder (DUD) nevertheless remain inadequately comprehended [11]. This research is intended to determine the function of lncRNA (HOTAIR), miR-206, and BDNF in heroin use disorder (HUD) in addition to analyzing and correlating the PI3K/AKT/mTORC1/Arc pathway expression. MiR-206 affects heroin use and BDNF expression, which is measurable through PCR. Since this pathway is new, we focused on it in our research and aimed to demonstrate its impact.

Dysregulated miRNAs in neural and cerebral tissues have been linked to various DUD-related neurobiological processes, such as synaptic plasticity [25–27]**.** In addition, it has been stated that several brain-enriched microRNAs, including miR-132 and miR-212, contribute to the progression of addiction through direct modulation of dendritic spine morphogenesis, synaptic remodeling, and drug-seeking behaviors [26]. Consequently, identifying specific miRNAs linked to addiction is critical in elucidating narcotics addiction mechanisms and additional clinical implications for finding innovative therapeutic targets for this substantial public health concern. Previous studies revealed that miRNAs are stable in cells and serum/plasma, suggesting they could be used as blood-based biomarkers in various disorders [28].

In this study, a significant increase in miR-206 expression is observed, which is consistent with the analysis of Gu W-J et al. [12], who stated steadily increased Let-7b-5p, miR-206, and miR-486-5p levels in heroin drug addicts. Similarly, Hsu et al. [29] reported miR-206 and miR-210 overexpression in 50 opioid use disorder (OUD) patients who were receiving methionine medical treatment.

HOTAIR expression downregulation is observed in this study. This is the first study to establish a connection between HOTAIR and HUD. Several studies have demonstrated a relationship between lncRNA HOTAIR and miR-206. HOTAIR negatively controlled miR-206 expression in medulloblastoma, colorectal, and breast cancers [15, 17, 18].

Similarly, the current study demonstrates that a negative correlation of statistical significance exists between the expression levels of HOTAIR and miR-206.

This is consistent with the findings of the research by Li et al. [16]**,** who found that HOTAIR specifically binds to miR-206, sponging it in squamous cell carcinoma. Furthermore, miR-206 prevented the PI3K/AKT signaling pathway. Additionally, the growth of HNSCC tumors in nude rodents was inhibited when HOTAIR was silenced, or miR-206 was overexpressed, confirming the regulatory function of HOTAIR on miR-206.

This study illustrates decreased BDNF levels in the sera of HUD patients, most probably due to increased miR-206. miR-206 has been identified to modulate BDNF in the hippocampus of a learning-deficient mouse model and an Alzheimer's disease (AD) animal model**.** In AD mouse models, antagonizing miR-206 in the cerebral ventricles boosted the brain levels of BDNF, enhanced their memory function, and increased hippocampal synaptic density and neurogenesis [30].

The relationship between miR-206 and BDNF has been investigated in some diseases like schizophrenia (SZ), depression, and alcoholism. SZ patients had downregulated BDNF expression that got elevated one month after antipsychotic treatment, along with decreased miR-206 expression [31]. In a depression model, increased miR-206-3p expression in the hippocampus was associated with a decreased hippocampal BDNF signaling cascade. The administration of AntagomiR-206-3p intranasally and genetic suppression of hippocampal miR-206-3p both induced an antidepressant response and downregulated miR-206-3p with the inclusion of the hippocampal BDNF-TrkB system [32].

Moreover, another study by Tapocik et al. [33] demonstrated that alcohol withdrawal after chronic exposure resulted in the increased expression of miR-206, which targeted BDNF, fostering an increase in the practice of self-administering alcohol. Tapocik et al. [33] not only demonstrated that miR-206 prevents the secretion of BDNF protein in cortical neurons (in vitro) and binds to and hinders the expression of the BDNF transcript (in vitro) and protein (in vivo), but additionally demonstrated the functional impact of miR-206 expression on alcohol-related conduct, where non-dependent rats revealed an escalation of alcohol self-administration associated with downregulation of BDNF upon miR-206 overexpression. The team further explored the miR-206-BDNF relationship by analyzing bioinformatics utilizing microRNA.org. Three conserved rno-miR-206 target sites were identified in the 3′-UTR of rat BDNF, which is complementary to the seed sequence of the miR-206 family of microRNAs.

Application of a dual luciferase reporter assay portrayed 60% BDNF repression by miR-206, based on the existence of two of the three rno-miR-206 target sites in rat BDNF 3′-UTR [33].

In addition, Shi et al. [34] studied how BDNF was the target gene of miR-206 by a dual-luciferase reporter system that demonstrated a reduced percentage of Renilla luciferase/firefly luciferase in the miR-206 and BDNF-WildType-3′UTR co-transfection groups. In contrast, a significant ratio elevation was observed in the BDNF-MutantType-3′UTR group. The team was studying the impact of Meridian massage on miR-206 and its subsequent effect on BDNF. Further verification was conducted by inducing miR-206 overexpression and then silencing in C2C12 myoblast cells. Gene and protein expression outcomes illustrated that traction tension-treated C2C12 myoblasts had decreased miR-206 expression. An overexpression of miR-206 immensely decreased BDNF expression, whose level was reinstated subsequent to treatment with traction tension, in contrast to miR-206 inhibition.

This study coincides with others like Zhang et al. [35] and Lu et al. [36], which demonstrated lower BDNF serum levels in heroin-dependent patients than in controls. Moreover, following 26 weeks of abstinence, BDNF levels rose, although they were still lower than controls [35, 36]**.** Contrary to that, research by Zhang et al. [20] found higher serum BDNF levels among heroin abusers than in controls. It is therefore worth mentioning that the genetic variant of BDNF '*Val66Met'* was previously associated with decreased serum BDNF along with a self-reported risk-taking propensity among heroin users [37]. However, the BDNF 'Val68Met' polymorphism was found to increase compulsive alcohol drinking in mice [38], and the BDNF rs6265, rs11030104, and rs10767664 were reported to be associated with decreased heroin addiction risk [39].

In addition, it was found that some miRNAs and their downstream targets illustrated altered expressions explicitly related to the BDNF 'Val66Met'. This suggested that the BDNF 'Val66Met' may intervene with specific psychiatric and cognitive phenotypes because of its ability to modify the affinity of miRNAs on their target-binding regions on the BDNF gene [40].

Synaptic plasticity malfunctioning is commonly found in addiction. This could be explained by impaired local translation since the experience-mediated alterations in synaptic strength are partly managed by the ability to translate mRNAs locally and rapidly at synaptic sites. mTORC1 (the mechanistic target of rapamycin complex 1) is a chief regulator of local dendritic and synaptic protein translation. Therefore, mTORC1 significantly functions in synaptic plasticity, learning, and memory [41]. mTOR upregulation in HUD is observed in this study, which is consistent with Ucha et al. [22], who aimed to evaluate the basic architecture of the complex intracellular network and describe the post-drug abuse changes in its components.

Moreover, the PI3K/AKT gene expression levels are reduced in the HUD group in this study. This aligns with the increase in miR-206 expression, as it inhibits PI3K/AKT by targeting c-Met, a receptor tyrosine kinase that stimulates the PI3K/AKT pathway. By inhibiting c-Met, miR-206 suppresses the stimulation of the PI3K/AKT pathway [42]. Recently, a functional enrichment analysis of the peripheral blood transcriptome of OUD participants' study recognized that PI3K/AKT signaling pathways were associated with OUD [43].

PI3/AKT negatively regulates mTOR. The PI3K/AKT/mTOR signaling pathway may be involved in the process of heroin relapse and addiction since AKT phosphorylation in the NAc may have a substantial impact on the induction of heroin-seeking behaviors [23].

In addition, post-mortem brain specimens from opiate users demonstrated that chronic heroin administration decreases levels of total IRS2 (insulin receptor substrate 2), phospho-AKT, and phospho-GSK3 (glycogen synthase kinase 3) in the rat ventral tegmental area but increases mTORC1 signaling [23, 44].

It is noteworthy that mTORC1 signaling is additionally associated with the mode of action of various classes of addictive substances, including psychostimulants, cannabis, and alcohol [11], even after a single alcohol injection [45], mTORC1 was proven to facilitate relapses to alcohol drinking [46]**.** Antagonizing mTOR in rodent models indicated the role of mTORC1 in alcohol-induced synaptic plasticity in D1 (dopamine D1 receptor-expressing neurons) neurons in the NAc shell [47]. It was suggested by Laguesse & Ron [11] that drugs of abuse induce maladaptive synaptic plasticity and arouse addiction-related behaviors by acting on and stimulating diverse downstream receptors and signaling pathways, eventually converging on mTORC1 [11].

Activity-regulated cytoskeleton-associated protein (Arc) is an essential brain-derived neurotrophic factor (BDNF) effector. It was previously stated that dendritic Arc synthesis was induced in hippocampal neurons activated with BDNF in culture. Rapamycin was found to inhibit this process [48]. A possible explanation is that BDNF, through the phosphorylation of the cAMP-responsive element-binding protein (CREB), upregulates CREB-target genes, including the Arc protein [49, 50].

A study on the differentially methylated CpG sites (DMSs) between heroin addicts and controls revealed that *Arc* is hypermethylated in heroin users [51].

Li et al. [16] investigated possible mechanisms underlying decreased cue-induced reinstatement of cocaine seeking in adolescent-onset self-administration as opposed to adult-onset self-administration (in drug addiction, cues formerly associated with drug use can produce craving and regularly trigger the resumption of drug-taking in individuals susceptible to relapse).

Despite similar intravenous levels of cocaine, it was observed that adolescent onset rats exhibited higher gene expression of Arc and BDNF in the prefrontal cortex (PFC) and nucleus accumbens (NAc) than adult-onset rats. This finding suggests that Arc and BDNF may play a safeguarding function against future drug-seeking behavior in adolescent-onset rats [52]. Interestingly, Arc is stated to physically modulate the dendritic spine density (DSD) and function [53], an effect antagonized by the inhibition of miR-206. The authors explained this by the elevated BDNF expression, which eventually increased Arc expression, DSD, and neurogenesis in the mouse hippocampus [54].

In an additional investigation involving cultured rat cortical neurons, the application of BDNF induced the phosphorylation of eukaryotic initiation factor 4E binding protein (4EBP1) and p70 ribosomal S6 kinase (p70S6K), the dephosphorylation of eukaryotic elongation factor 2 (eEF2), and an upregulation of Arc expression. BDNF inhibitors impeded the activity of the PI3K-Akt-mTOR pathway [55, 56].

## Conclusion

HOTAIR and miR-206 may have an impact on the expression of synaptic plasticity genes Arc and BDNF by analysing the expression of the PI3-AKT-mTOR pathway [12, 33]**.** Compared with controls, HUD patients portray higher miR-206 expression, associated with decreased HOTAIR expression, lower serum BDNF levels, and downregulated Arc expression. A regulatory role of HOTAIR on miR-206 in HUD is suggested, possibly via the PI3/AKT/mTOR pathway. This might participate in neurochemical and behavioral plasticity associated with relapses and addictive behavior. These findings could develop new biomarkers for HUD relapses and potential RNA therapeutics in clinical and forensic medicine. Further research with a larger sample size is recommended.

## Data Availability

No data associated in the manuscript.
